# Biologics in the Treatment of Lupus Erythematosus: A Critical Literature Review

**DOI:** 10.1155/2019/8142368

**Published:** 2019-07-18

**Authors:** Dominik Samotij, Adam Reich

**Affiliations:** Department of Dermatology, University of Rzeszow, ul. Fryderyka Szopena 2, 35-055 Rzeszow, Poland

## Abstract

Systemic lupus erythematosus (SLE) is a chronic autoimmune inflammatory disease affecting multiple organ systems that runs an unpredictable course and may present with a wide variety of clinical manifestations. Advances in treatment over the last decades, such as use of corticosteroids and conventional immunosuppressive drugs, have improved life expectancy of SLE sufferers. Unfortunately, in many cases effective management of SLE is still related to severe drug-induced toxicity and contributes to organ function deterioration and infective complications, particularly among patients with refractory disease and/or lupus nephritis. Consequently, there is an unmet need for drugs with a better efficacy and safety profile. A range of different biologic agents have been proposed and subjected to clinical trials, particularly dedicated to this subset of patients whose disease is inadequately controlled by conventional treatment regimes. Unfortunately, most of these trials have given unsatisfactory results, with belimumab being the only targeted therapy approved for the treatment of SLE so far. Despite these pitfalls, several novel biologic agents targeting B cells, T cells, or cytokines are constantly being evaluated in clinical trials. It seems that they may enhance the therapeutic efficacy when combined with standard therapies. These efforts raise the hope that novel drugs for patients with refractory SLE may be available in the near future. This article reviews the current biological therapies being tested in the treatment of SLE.

## 1. Introduction

**Figure 1:**
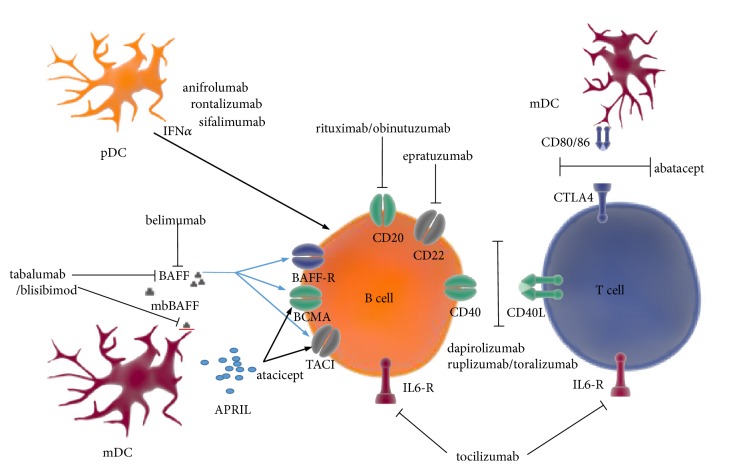
Targets for biological therapies in systemic lupus erythematosus (modified from [14]). APRIL: a proliferation-inducing ligand; BAFF: B cell-activating factor belonging to the TNF family; BAFF-R: BAFF receptor; BCMA: B cell maturation antigen; CD40L: CD40 ligand; CTLA4: cytotoxic T lymphocyte associated protein 4; IFN*α*: interferon alpha; IL6-R: interleukin 6 receptor; mbBAFF: membrane-bound BAFF; mDC: myeloid dendritic cell; pDC: plasmacytoid dendritic cell; TACI: transmembrane activator-1 and calcium modulator and cyclophilin ligand interactor.

Systemic lupus erythematosus (SLE) is a chronic multisystemic inflammatory disease, in which tissue injury is a consequence of immune dysregulation and tolerance loss to nuclear self-antigens. Conventional treatment modalities for SLE have allowed an improvement of survival; however, their use is associated with a significant risk of toxicity and a wide range of morbidities. The research conducted in recent years has significantly contributed to the extension of our knowledge on SLE immunopathology and provided important data on the potential targets of novel therapies (Figure 1). Efforts are being constantly made to develop drugs with a more selective mode of action, targeting the strictly defined immunological targets relevant to SLE, while at the same time allowing the reduction of adverse events (AEs) of the previously used drugs. In this review we summarized the available results of clinical trials with selected biological drugs in SLE (Table 1).

## 2. B Cell-Targeted Therapies

Increased activity of B cells has been demonstrated both in animal models and in human with SLE [1, 2]. SLE is characterized by abnormal activation and differentiation of B cells as well as defective elimination of autoreactive B cells [3]. Increased B cell activity results in polyclonal hypergammaglobulinemia and the production of numerous autoantibodies, in particular those recognizing insoluble (e.g., histones or native DNA) and soluble (extractable) nuclear antigens (ENA, e.g., Smith or snRNP antigen) [4]. The importance of B cells in SLE physiopathology is related not only to their ability to produce autoantibodies, but also to the capability of presenting autoantigens to T cells through the BCR (B cell receptor) and cytokine secretion and modulation of dendritic cell (DC) activity [5, 6]. An ideal B cell-targeted drug should therefore eliminate pathogenic B cells and/or trigger B cell expansion and promote the function of protective cells.

The protein called BLyS (B lymphocyte stimulator), also known as BAFF (B cell-activating factor belonging to the tumor necrosis factor family) or TNFSF13B (the tumor necrosis factor ligand superfamily member 13B), belongs to the superfamily of TNF ligands [7, 8]. BLyS is a soluble membrane protein that is secreted by monocytes, neutrophils, and T cells and DCs [9]. BLyS binds only to the receptors located on the surface of B cells. The following 3 types of receptors for BLyS were identified: BR3 (BLyS receptor 3), TACI (transmembrane activator-1 and calcium modulator and cyclophilin ligand interactor), and BCMA (B cell maturation antigen). BR3 binds only BLyS whereas TACI and BCMA may also bind APRIL (a proliferation-inducing ligand). BLyS binds with much higher affinity to BR3 than to BCMA, whereas APRIL has a greater affinity for BCMA and little or no binding capacity for BR3. Biological activity of BLyS thus depends to the greatest extent on BR3 [10]. BLyS plays an important role in the differentiation, activity, and survival of B cells [11]. Increased concentration of BLyS affects the survival of B cells that produce autoantibodies by inhibiting their apoptosis [12]. BLyS-targeted therapy selectively reduced the number of CD20+ lymphocytes and short-lived plasma cells as well as anti-double-stranded DNA (anti-dsDNA) antibody titers in patients with SLE; however, this therapy did not affect memory B cells and long-lived plasma cells [13]. The discovery of the crucial role of BLyS in the pathogenesis of SLE, as a cytokine responsible for survival, activation, and differentiation of B cells, has led to the development of new molecules directed against BAFF, which have been demonstrated to be promising agents in clinical trials.

### 2.1. B Cell Growth and Survival Factors Inhibitors

#### 2.1.1. Belimumab

Belimumab is the first drug to be registered for the treatment of SLE since 1955, when hydroxychloroquine was repurposed and approved [15]. Belimumab in intravenous (IV) formulation administered at a dose of 10 mg/kg body weight (BW) on days 0, 14, and 28 and then every 28 days was approved by the Food and Drug Administration (FDA), the European Medicines Agency (EMA), and the National Institute for Health and Clinical Excellence (NICE) [16, 17]. Belimumab is indicated for the treatment of adult patients with seropositive SLE with active cutaneous or joint disease, who do not respond to standard treatment, excluding patients with lupus nephritis (LN) and severe central nervous system (CNS) involvement [18].

Belimumab is a human IgG1*λ* monoclonal antibody that has the ability to bind the soluble BLyS receptor preventing survival of B cells; it further reduces the differentiation of B lymphocytes into plasma cells that produce immunoglobulins (Igs). BAFF-neutralizing therapy with belimumab reached the primary end points in two large phase III clinical trials conducted on over 1,600 SLE patients [19, 20]. The results of these studies were the basis for the registration of this drug by the FDA.* Post hoc* analyses of these trials suggested a significant benefit from belimumab treatment, especially in patients with more active disease, treated with higher doses of corticosteroids (CS), with the presence of anti-dsDNA antibodies and low serum levels of C3 and C4 complement components [21]. The initial favorable observations on the use of belimumab in SLE had already been made based on phase I studies and they were confirmed in the subsequent trials [22]. A total of 449 patients included in the second phase study received the drug at a dose of 1, 4, and 10 mg/kg BW or placebo as an addition to standard therapy on days 0, 14, and 28 and then every 28 days for 52 weeks. A serologically active group (71.5% of patients with detectable antinuclear or anti-dsDNA antibodies) was isolated from the patients studied. In these individuals, a statistically significant decrease in the SELENA-SLEDAI (Safety of Estrogens in Lupus Erythematosus National Assessment-Systemic Lupus Erythematosus Disease Activity Index) score, a reduction in PGA (Physician Global Assessment) score, and improvement of the quality of life (QoL) were found. The study also showed that belimumab caused depletion of about 60-70% of CD20+ cells and decreased the number of naïve and activated B cells and plasmacytoid cells. Reduction of anti-dsDNA antibody titres by 29% and reduction of the total Ig levels were also observed. At the same time, a favorable safety profile was demonstrated as the number of AEs (including serious AEs and infections) in patients receiving the drug was similar to the number of AEs in the placebo group [23].

Phase III clinical trials (BLISS-52 and BLISS-76) were carried out according to a similar protocol, but they were undertaken in different geographical regions. Patients with serologically positive SLE were randomized to one of three groups: the placebo group and the belimumab group at a dose of 1 mg/kg BW or at a dose of 10 mg/kg BW. The drug was administered on days 0, 14, and 28 and then every 28 days. In both studies, the primary end point, i.e., the proportion of patients achieving SRI-4 (Systemic Lupus Erythematosus Responder Index 4), was significantly higher among those receiving belimumab at a dose of 10 mg/kg BW than placebo (58% vs. 44% in BLISS-52 and 43% vs. 34% in BLISS-76). A significantly higher proportion of patients treated with belimumab exhibited an improvement in musculoskeletal and mucocutaneous symptoms [24]. There was also a positive effect of the drug on immunological indicators of disease activity (reduction of Ig concentration and increase in complement components, reduction of autoantibody production), as well as improvement of QoL and level of fatigue [25, 26]. In the BLISS-52 study, a significantly higher number of patients in the 10 mg/kg BW arm were eligible for prednisone dose tapering by 50% in weeks 24-52 compared to those receiving placebo, although such a prominent CS-sparing effect was not observed in the BLISS-76 study. In addition, a study using belimumab given as a subcutaneous (SC) injection of 200 mg every 7 days (BLISS-SC study) combined with standard treatment regimen in patients with disease activity score ≥8 points according to SELENA-SLEDAI showed SRI-4 response at week 52 to be significantly higher in the active group than in the placebo group (65% and 47%, respectively). Moreover, patients treated with belimumab had a 62% lower risk of severe exacerbation compared to the placebo group [27]. A recent network meta-analysis comparing the efficacy and safety of belimumab administered as an IV or SC injection has demonstrated that IV belimumab at a dose of 10 mg/kg BW and SC belimumab 200 mg had the highest probability, based on surface under the cumulative ranking curve, of being the best treatment for achieving the SRI response at week 52. Furthermore, it was concluded that both of aforementioned belimumab dosage regimens combined with standard therapy were efficacious interventions for active SLE and were not associated with a significant risk of SAEs [28]. Despite relatively high costs of therapy, pharmacoeconomic studies carried out in Greece, Spain, and Italy have shown that belimumab treatment is profitable [29–32]. The European expert panel guidelines published in 2017 recommended the use of belimumab in treatment-resistant and CS-dependent SLE, in the absence of renal and CNS involvement or severe autoimmune thrombocytopenia [33]. A frequent indication for adding belimumab to the standard treatment is also the constant progression of immunological markers of disease activity [34]. Experts suggest that the efficacy of the drug should be evaluated 16 weeks after the first drug administration at the earliest. Lack of efficacy after 6 months of treatment is an indication for the drug discontinuation [35].

#### 2.1.2. Tabalumab

Tabalumab is a fully human IgG4 monoclonal antibody that neutralizes BAFF (both soluble and membrane-bound forms) but does not bind to APRIL [36, 37]. This biologic was directly studied in two 52-week phase III randomized double-blind placebo-controlled clinical trials in patients with active SLE, but without severe renal involvement and neuropsychiatric symptoms [38, 39]. The first study (ILLUMINATE-1) included 1,164 patients with moderate-to-severe SLE (SELENA-SLEDAI score ≥6 points). At week 52, the proportion of patients who achieved the primary end point (SRI-5) was not significantly higher among those receiving the drug than among those on placebo. Secondary end points (including time to the first severe flare of the disease, CS-sparing effect, and reduction of fatigue level) were also not met. Nonetheless, tabalumab significantly affected the immunological parameters of the disease; marked reductions in the serum concentration of anti-dsDNA antibodies, an increase in serum levels of the C3/C4 complement components, and a reduction in the total B cell count, Ig concentration, and BAFF levels were observed. The frequency of AEs and mortality did not differ between those treated and those receiving placebo.

The second study (ILLUMINATE-2) concerned patients with active SLE (n = 1,124). The primary end point (percentage of patients who achieved SRI-5) was met at week 52 in the 120 mg group every 2 weeks (38% vs. 28% in the treatment group and placebo, respectively; p = 0.002). On the other hand, no secondary end points have been met. In* post hoc* analyses of the efficacy of tabalumab using the same clinical response rate that was used in the belimumab phase III trials (i.e., SRI-4 instead of SRI-5), a statistically significant 11% difference in the response rate in favor of the group of patients on tabalumab was observed compared to placebo receivers [40]. Nevertheless, further work on tabalumab has been halted.

#### 2.1.3. Blisibimod

Blisibimod is a fusion protein composed of four BAFF-binding domains fused to the N-terminal Fc fragment of human IgG1 Ig. This biologic agent is a potent BAFF inhibitor administered in the form of SC injections [41]. In a double-blind, randomized, placebo-controlled clinical trial (PEARL-SC), 547 patients with serologically active SLE and SELENA-SLEDAI score ≥6 points were randomized to 3 different doses of blisibimod or placebo. At week 24, the highest dose group (200 mg once weekly) had a significantly higher SRI-5 response rate than the placebo group. Blisibimod treatment was associated with a significant improvement in biomarkers: lowering of anti-dsDNA antibodies, increase in serum C3/C4 concentrations, and reduction of B cell number. The drug was well tolerated in all doses [42].

The results of a multicenter, placebo-controlled, phase III randomized, double-blind study (CHABLIS-SC1) conducted on a group of 442 seropositive SLE patients with persistent high disease activity (SELENA-SLEDAI ≥10 points) despite use of CS and other standard treatments did not show significant differences in the frequency of the primary end point of the study (SRI-6) between blisibimod and placebo receivers (47% vs. 42%, respectively). The secondary end points (SRI-4 and SRI-8) were also not reached. Despite disappointing results in terms of study end point measures, significant beneficial changes in the number of B cells, the concentration of Igs, and complement components were demonstrated similarly to the phase IIb clinical trial [43].

The continuation of CHABLIS-SC1 was CHABLIS7.5 study (also known as CHABLIS-SC2), allowing the recruitment of patients with renal involvement, which included patients with severe SLE (SELENA-SLEDAI score ≥10 points), the presence of anti-dsDNA antibodies in serum and depressed levels of complement components despite the use of CS in a dose compatible with the standard-of-care. However, this study was terminated prematurely [44].

#### 2.1.4. Atacicept

Atacicept (TACI-Ig) is a fully human recombinant fusion protein that neutralizes both BAFF and APRIL [45]. This agent is an inhibitor of both soluble and membrane-associated BAFF. Placebo-controlled phase Ib clinical trial with dose escalation in patients with SLE showed the biological efficacy of atacicept as measured by reduction in peripheral B cell counts and dose-related Ig levels [46]. Subsequently, a 52-week phase II/III study with atacicept (150 mg SC 2x weekly for 4 weeks, then 1x weekly), double-blind, randomized, placebo-controlled in patients with active LN who received an additional high dose of CS and mycophenolate mofetil (MMF) (3g/day) was set up. Unfortunately, this trial was discontinued shortly after initiation due to an unexpected severe decrease of serum IgG levels and serious infections [47].

Another double-blind phase II/III trial, with randomization and placebo control, was performed on a group of 461 patients. Participants of this study with active SLE according to the BILAG (British Isles Lupus Assessment Group) index (≥1 item in the BILAG A and/or B domain), previously treated with CS with gradual dose reduction for 10 weeks who reached the BILAG C or D domain, were randomized in a 1:1:1 ratio to placebo and two doses of atacicept (75 mg or 150 mg 2x per week for 4 weeks, then 1x per week for 48 weeks). The primary end point, defined as a significantly decreased proportion of patients who developed a new flare from BILAG A or BILAG B domain scores, was not met in the 75 mg arm; time to exacerbation was also not significantly prolonged between the 75 mg treatment arm and the placebo arm. Treatment of patients in the 150 mg arm was not further continued due to serious AEs. Administration of the drug in both doses resulted in an improvement with respect to the immunological parameters of the disease, as well as a reduction of the total Ig concentrations [48]. Opposingly, the results of the 24-week phase IIb randomized placebo-controlled trial (ADDRESS II) for patients with active SLE (SLEDAI-2K score ≥6 points) receiving standard therapy completed in 2016 did not confirm the higher incidence of serious AEs with both doses of atacicept (75 mg and 150 mg SC) compared to placebo. The percentage of treatment responses expressed by SRI-4 was higher among patients receiving the drug than placebo. Interestingly, patients with high baseline disease activity (SLEDAI-2K score ≥10 points) did not benefit from the therapy [49].

*Post hoc* analysis showed a relationship between the dose administered and the reduction of Ig concentrations and the frequency of exacerbations. According to the original hypothesis, elevated BLyS and APRIL levels were associated with better response, which was expressed primarily by a more pronounced decrease in the frequency of flares [50].

### 2.2. Immunological Tolerance-Inducing Agents (Tolerogens)

#### 2.2.1. Abetimus

Abetimus (abetimus sodium or LJP-394) is a synthetic molecule consisting of deoxynucleotide-like molecules in a tetrameric form, mimicking the dsDNA-containing immune complexes. It has the ability to bind the Ig receptor to native DNA on the surface of B cells by cross-reacting, which affects intracellular transmission leading to anergy or apoptosis of these cells. This further induces B cell tolerance to own antigens derived from native DNA (the so-called tolerogenic therapy) [51]. Abetimus is a highly specific molecule consisting of over 97% of native DNA; hence it interacts only with molecules that recognize native DNA. The action of abetimus in reducing the concentration of circulating anti-dsDNA antibodies persists long after its administration, even when it is already absent in the circulation [52].

Despite drug efficacy having been demonstrated in a randomized, double-blind, placebo-controlled phase II/III trial in LN individuals (reduction of exacerbation frequency and prolongation of time to the first flare), a phase III study in patients with anti-dsDNA-positive SLE did not confirm its effectiveness with respect to reduction of the frequency of SLE flares, even in a subgroup of patients with high-affinity antibodies to the DNA epitope found in abetimus [53, 54]. Despite that, this drug reduced the concentration of anti-dsDNA antibodies, caused an increase in serum C3 complement component, and was well tolerated. Shortly thereafter, a large number of patients (n = 943) were included in the subsequent randomized, double-blind, placebo-controlled phase III clinical trial (ASPEN) employing a higher dose of drug with a high risk of LN flare. This study, however, was prematurely interrupted based on a preliminary analysis that did not show any benefits of using this molecule [55].

### 2.3. Monoclonal Antibodies Targeting B Cell Surface Antigens

#### 2.3.1. Anti-CD20 Monoclonal Antibodies

*(1) Rituximab*. Rituximab (RTX) is a chimeric mouse-human monoclonal antibody that selectively binds to the CD20 transmembrane antigen present on the surface of B cells (from pre-B cells to memory B cells). RTX selectively binds CD20-positive cells which results in cell-cycle arrest and eventually leads to apoptosis. These cells are further destroyed via complement-dependent lysis, antibody-dependent cellular cytotoxicity, and phagocytosis; these mechanisms depend on the Fc portion of the antibody binding to Fc*γ*Rs on immune cells [56]. B cell dysfunction causes disruption of their differentiation towards plasma cells and, as a consequence, a reduction in the production of pathogenic autoantibodies: anti-dsDNA and anti-nucleosome antibodies. Inhibition of CD20-positive cells induces depletion of all mature B cell forms but has no effect on plasma cells. Repopulation of B cells, which usually occurs 6-9 months after administration of RTX, predominantly involves a subset of naïve or antigenically inexperienced transitional B cells [57]. Repeated RTX administrations may cause hypogammaglobulinemia [58].

The first reports on the effectiveness of RTX in patients with SLE come from 2002 [59]. In subsequent studies, the efficacy of this drug was confirmed in patients with SLE and LN, with joint and mucocutaneous symptoms, serositis, cytopenia, and neurological involvement [60]. Analysis of 188 cases of RTX use in SLE in small, uncontrolled studies confirmed its effectiveness in this indication [61]. However, two large-scale, double-blind, randomized, placebo-controlled studies in patients with nonrenal SLE (EXPLORER, phase II/III) and LN (LUNAR, phase III) failed to meet their major end points [62, 63].

The first trial included 257 patients with moderate-to-high activity extrarenal disease (defined as BILAG A in ≥1 domain or BILAG B in ≥2 domains) persisting despite treatment with one immunosuppressive drug [62]. Notably, it was later emphasized that major clinical end point of this trial defined as achieving BILAG C scores or better in all assessed organs is difficult to obtain in “real life” setting [64].

The second clinical trial evaluated the efficacy of RTX in patients with active LN. In addition to RTX, the patients received CS and MMF (at a dose of 2 g/day). At week 52, no statistically significant differences were found in the number of patients achieving primary and secondary end points between RTX and placebo arms. However, the percentage of responders in the RTX arm was higher compared to placebo (57% vs. 46%). Greater improvement with respect to SLE-specific immunological markers, such as the concentration of anti-dsDNA antibodies and C3/C4 complement component levels, was also observed in the group of patients who received RTX. Neutropenia, leukopenia, herpes zoster, and opportunistic infections, as well as hypotension, fever, skin rash, and other AEs directly related to the IV infusion, were quantitatively more frequent in the RTX group [63]. Seeking explanations for the disappointing results of the LUNAR study, it is often underlined that background immunosuppressive regimen with repetitive infusion of 1 g methylprednisolone and high-dose oral MMF (as required by the protocol) could mask the effectiveness of RTX [65].

The results of the clinical trials discussed above did not show any significant benefits of using RTX in patients with SLE and somewhat diminished its importance in the treatment of this disease. Nevertheless, its frequent off-label use in daily clinical practice to control treatment-resistant SLE symptoms (including LN) should not be overlooked [66–70]. In patients with LN, RTX has been often given in combination with CS and/or other immunosuppressants, such as cyclophosphamide (CY), MMF, azathioprine, and methotrexate [68, 71, 72]. Data from the French AutoImmunity and Rituximab registry (n = 136) on RTX-treated patients with SLE showed 71% overall response rate by the SELENA-SLEDAI assessment; articular, mucocutaneous, renal, and hematologic improvements were noted in 72%, 70%, 74%, and 88% of patients, respectively. Interestingly, the efficacy of RTX monotherapy was not significantly different from that of RTX in combination with immunosuppressants [66].

In another clinical trial performed in the pediatric population, the RTX regimen without long-term oral CS showed efficacy in patients with LN [72]. Two doses of RTX (1 g/dose at 14-day intervals) and 2 IV methylprednisolone pulses (500 mg on days 1 and 15) were given to patients (n = 50) in combination with MMF maintenance therapy. A total and partial response at week 52 was obtained in 52% and 34% of participants, respectively. On the basis of promising results of the described therapeutic regimen, a similar clinical trial was commenced aiming to compare the regimen above with a conventional oral prednisone dose of 0.5 mg/kg BW/day in combination with MMF, but without RTX (RITUXILUP study) [73].

A substantial variability of B cell depletion rate following anti-CD20 therapy is described, which may influence the response and the frequency of SLE flares during RTX treatment. The phenomenon of poorer response to the drug concerns in particular those patients who exhibit more rapid repopulation of memory B cells and plasmablasts [74]. Increased baseline BLyS level and development of RTX-neutralizing antibodies are associated with worse clinical response [75, 76]. Due to the frequently observed increase in BLyS level after RTX treatment of potential clinical relevance, a proof-of-concept study was initiated to evaluate the sequential administration of belimumab after induction therapy with CY combined with RTX in patients with LN (CALIBRATE) [77, 78]. A clinical trial aimed at assessing the efficacy of belimumab in combination with RTX began recruitment in early 2018 [79].

RTX, as a chimeric antibody, can cause hypersensitivity reactions which have been reported in 10-15% of patients with SLE [80]. Another significant issue in the treatment of SLE patients with RTX seems to be a relatively high frequency of so-called human antichimeric antibodies (HACAs). The clinical relevance of HACA formation in RTX-treated patients is not yet fully elucidated [76].

*(2) Ocrelizumab*. Ocrelizumab is a fully humanized (90%) anti-CD20 monoclonal antibody synthesized to reduce immunogenicity and increase tolerability [81]. Ocrelizumab is the first disease-modifying treatment registered for patients with early primary progressive multiple sclerosis [82]. In vitro studies of this molecule have demonstrated increased antibody-dependent cell-mediated cytotoxicity and lower complement-dependent cytotoxicity compared to RTX [83]. Two phase III clinical trials evaluating this agent in patients with SLE have been completed; the first study examined the efficacy of ocrelizumab in SLE without renal involvement (BEGIN), while the second one recruited patients with LN (BELONG). The BEGIN study was terminated prematurely due to the lack of response.

In BELONG trial, patients with active proliferative LN received ocrelizumab or placebo on the background of either MMF or CY in the so-called Euro-Lupus regimen (500 mg IV CY every 2 weeks to a total of 6 doses and azathioprine). The trial was also terminated early due to a significant increase in serious infections (in the MMF + ocrelizumab group). The efficacy analysis showed a nonstatistically significant numerical superiority of ocrelizumab over placebo; complete renal response in the ocrelizumab treatment groups (67%) was achieved nonstatistically more frequently than in the placebo group (55%). The response rate was higher in patients receiving ocrelizumab with CY than in those on ocrelizumab with MMF. Severe infections were statistically more frequent within the MMF group [84, 85].

*(3) Ofatumumab*. Ofatumumab is a fully human IgG1*κ* monoclonal antibody that binds to a unique epitope on the human CD20 molecule expressed on the surface of B cells; this agent has low immunogenicity [86]. Ofatumumab was often used off-label to treat SLE, in particular in responders to RTX who developed hypersensitivity to this drug [87–89]. In clinical trials conducted in chronic lymphocytic leukemia and rheumatoid arthritis populations, no significant induction of anti-drug antibodies was observed [90, 91]. Formal clinical trials on the use of this drug in SLE are expected.

*(4) Obinutuzumab*. Obinutuzumab is a novel fully humanized glycoengineered IgG1 type II monoclonal antibody against CD20, which is used in the treatment of patients with chronic lymphocytic leukemia [92]. In vitro study showed its greater capacity for B cell depletion compared to RTX [93]. A 52-week phase II trial in patients with LN is ongoing with complete renal response as a primary end point [94].

### 2.4. Anti-CD22 Monoclonal Antibodies

#### 2.4.1. Epratuzumab

Epratuzumab is a recombinant, humanized IgG1 monoclonal antibody directed selectively against the CD22 antigen on the surface of mature B cells; CD22 is a B cell-specific surface antigen involved in the modulation of BCR signaling, cellular activation, and B cell survival [95]. Epratuzumab and RTX display distinct modes of action; epratuzumab acts as an immunomodulatory nondepleting agent, while RTX is an acutely cytotoxic molecule [96]. In vitro, epratuzumab has been shown to rapidly induce a significant reduction of CD22, CD19, CD21, and CD79b on the surface of B cells in both Fc-dependent and Fc-independent mechanisms. The functional consequence of binding epratuzumab to CD22 is inhibition of B cell proliferation and reduced expression of adhesion molecules and the synthesis of proinflammatory cytokines, such as interleukin 6 (IL-6) and TNF-*α* [97]. Epratuzumab is unable to induce complement-dependent cellular cytotoxicity; therefore it is safer than RTX with no infusion reactions reported so far. In clinical studies, epratuzumab led to a modest reduction in peripheral B cells without significant effect on T cells, autoantibody titres, and Ig levels after prolonged therapy [98–100].

EMBLEM was a phase IIb double-blind, randomized, placebo-controlled trial evaluating epratuzumab in patients with moderate-to-severe SLE (no severe neuropsychiatric SLE and LN patients were included in the study) [100]. At week 12, the overall proportion of responders was insignificantly higher in all groups of patients treated with epratuzumab. The frequency of AEs and serious AEs (including infusion reactions) was comparable in all patient groups. The study was later extended to an open-label phase for a follow-up period of 3.2 years which showed sustained improvements in disease activity and QoL. Serious infections developed in 6.9% of those treated; however the average dose reduction of CS was 50% at week 108 [101].

Subsequently, two phase III randomized, double-blind clinical trials with epratuzumab (EMBODY 1 and EMBODY 2) were performed, which recruited patients with moderate-to-severe seropositive SLE (≥6 points in SLEDAI-2K and ≥1 A domain and ≥2 in the mucocutaneous, musculoskeletal, or cardiorespiratory domains in BILAG 2004) despite the use of standard therapy, including mandatory treatment with CS. Patients with BILAG A in the renal and neuropsychiatric domains were not included in this study. Patients received either epratuzumab in one of two dosing schemes (600 mg every week or 1,200 mg every other week) or placebo. All patients maintained their previous background SLE therapies. The primary end point was the degree of response based on BICLA (BILAG-based Combined Lupus Assessment), a composite index based on BILAG. The study included a large number of patients (n = 1574); unfortunately, no statistically significant difference in end points (both primary and secondary) between the groups receiving the drug and placebo was found. The frequency of AEs was similar across all study arms. Neither the primary outcome nor any of the secondary outcomes were achieved in the aforementioned trials; therefore epratuzumab is no longer being developed for SLE [102].

## 3. Type I Interferon-Targeted Therapies

Activation of type I interferon (IFN) system is considered to be a key player in SLE immunopathogenesis [103–105]. Cell signalling of all type I IFNs, i.e., IFN*α*, IFN*β*, IFNɛ, IFN*κ*, and IFN*ω*, is mediated by the so-called IFNAR (type I IFN-*α*/*β*/*ω* receptor), resulting in IFN-stimulated gene transcription, also referred to as IFN gene signature [106]. It is therefore believed that IFNAR blockade may reverse some of the immunological imbalance developed by SLE patients [107].

IFN*α* is produced by many cell types, yet its synthesis by plasmacytoid DCs in particular is of the greatest importance from the point of view of the pathogenesis of SLE. In SLE, production of type I IFNs by plasmacytoid DCs is induced by complexes consisting of DNA/RNA-containing autoantigens through Fc*γ*R-dependent internalization of these complexes and activation of the toll-like receptor 7 (TLR-7) and TLR-9 [108]. IFN*α* promotes DC formation, activation of T cells, and production of autoantibodies by B cells [109]. Elevated levels of IFN*α* and IFN-dependent cytokines, as well as expression of IFN-regulated genes, correlated with serologic disease activity markers, such as anti-dsDNA antibodies, complement components, and IL-10 levels [110–116]. In addition, polymorphisms of many components of IFN-dependent signaling pathways were shown to be associated with higher susceptibility to SLE [117].

### 3.1. Rontalizumab

Rontalizumab is a human monoclonal IgG1 antibody that neutralizes all known IFN*α* subtypes but does not bind to IFN*β* or IFN*ω*. A phase I clinical trial established the safety and efficacy of the molecule in reducing the expression of IFN-regulating genes [118]. Immediately thereafter, a phase II clinical trial was conducted on a group of 238 patients with moderate-to-severe SLE (defined as BILAG A in ≥1 domain or BILAG B in ≥2 domains) and no renal involvement. Prior to its initiation, all background therapy was discontinued except for hydroxychloroquine, and the dose of prednisone (or equivalent) was tapered to ≤10 mg/day by week 8. At week 24, no significant difference in treatment response was seen based on BILAG and SRI between patients treated and those receiving placebo. The drug was well tolerated and no significant increase in the frequency of infections was observed [119]. Disappointingly, due to a lack of efficacy, further work on this molecule has been stopped.

### 3.2. Sifalimumab

Sifalimumab is a fully humanized IgG1*κ* monoclonal antibody that has the ability to bind and neutralize most of the 13 known IFN*α* subtypes. Two phase I clinical trials demonstrated the safety of this molecule in patients with seropositive SLE with moderate-to-high activity [120, 121].* Post hoc* analysis of the efficacy showed that the clinical effectiveness of the drug was better in patients with high initial expression of IFN gene signatures. Phase II clinical trial was conducted on a relatively large group of patients (n = 431) with active SLE (SLEDAI ≥6 points, BILAG A in ≥1 domain, and BILAG B in ≥2 domains, PGA ≥1); patients with CNS involvement were not included in the study. At week 52 a better response (measured by the percentage of SRI-4 improvement) was observed in the groups treated with all 3 doses of sifalimumab (200/600/1,200 mg) than in the placebo group (59.8% vs. 45.4%; p = 0.03). There was also a skin score improvement expressed in the reduction in CLASI (Cutaneous Lupus Erythematosus Disease Area and Severity Index) and a reduction in tender and swollen joint counts. Similarly to the observations from the previous study, patients with high baseline IFN gene signatures responded better. Sifalimumab was safe and well tolerated [122]. Interestingly, this drug did not affect immunological parameters of SLE activity, such as the concentration of anti-dsDNA antibodies and C3/C4 complement component titers in the serum. Despite confirming that blockade of type I IFNs is an effective measure to treat SLE and demonstrating the clinical efficacy of sifalimumab, the sponsor did not continue the development of this molecule.

### 3.3. Anifrolumab

Anifrolumab is a fully human IgG1*κ* monoclonal antibody that binds to IFNAR I by inhibiting the activity of all type I IFNs, including IFN*α*, IFN*β*, IFN*ε*, IFN*κ*, and IFN*ω* [123, 124]. Phase I clinical trial showed a greater and more prolonged suppression of IFN gene signatures in patients with SLE receiving anifrolumab compared to those using sifalimumab [125]. A subsequent 48-week phase IIb clinical trial was conducted with a double-blind, randomized, and placebo-controlled study in patients with nonrenal SLE who were not responding adequately to standard treatments. Participants were randomized to one of two groups receiving anifrolumab IV at different doses (300 mg or 1,000 mg) or to a placebo group. The primary end point was to achieve an SRI-4 response and maintain the prednisone dose at 10 mg/day or lower by day 169; it was met by a significantly larger proportion of patients treated with 300 mg of anifrolumab than by placebo receivers (34.3% vs. 17.6%; p = 0.01, respectively). As expected, improvement in relation to the achievement of the primary end point as well as secondary end points (improvement of cutaneous manifestations, CS-sparing effect) was more strongly expressed in patients with high IFN gene expression. The drug was well tolerated [126]. Encouraging results of the above described clinical trial enabled the planning of further phase II and III trials for patients with SLE and LN [127–130].

## 4. Cytokine Targeting Treatments

### 4.1. Tumor Necrosis Factor-Alpha (TNF-*α*) Inhibitors

The role TNF-*α* plays in SLE pathogenesis remains controversial. On the one hand, the level of this cytokine is elevated in serum and renal biopsy samples of patients with active disease; on the other hand, it is possible to induce antinuclear antibodies, and even full-blown SLE, with TNF-*α* antagonists [131, 132]. The results of a pilot clinical trial evaluating infliximab in SLE conducted on a small group of patients suggested that anti-TNF-*α* antibodies could be used in the treatment of LN, as well as refractory lupus arthritis and cutaneous manifestations of the disease. However, the appearance of antinuclear, anti-dsDNA, and anti-phospholipid antibodies was observed in rheumatoid arthritis patients [133, 134]. In view of the above, TNF-*α* blocking molecules are currently not recommended for the treatment of SLE and confined to heavily refractory cases only.

### 4.2. Interleukin-6 Inhibitors

#### 4.2.1. Tocilizumab

Tocilizumab is a humanized IgG1 monoclonal antibody directed against both soluble and membrane-bound IL-6 receptors [135]. IL-6 is a proinflammatory cytokine involved in the differentiation of B cells into plasma cells and T cells into effector T cells. IL-6, secreted mainly by macrophages and T cells, acts synergistically with type I IFN [136]. It is suggested that IL-6 may be the key driver of B cell hyperactivity in SLE and may mediate tissue damage in the course of the disease [137]. The increased levels of this cytokine in serum, renal, and skin biopsy samples in patients with SLE positively correlated with disease activity and serum anti-dsDNA antibodies titers [138, 139]. In a phase I clinical trial that assessed the efficacy of tocilizumab in SLE, there was a significant improvement in clinical parameters, in particular in the joints, as well as in serum anti-dsDNA antibodies. The incidence of AEs, primarily dose-dependent neutropenia and mild-to-moderate infections, did not significantly differ from those documented in rheumatoid arthritis trials [140].

#### 4.2.2. Sirukumab

Sirukumab is another IL-6 blocking monoclonal antibody that binds directly to this cytokine. In phase I, placebo-controlled trial in patients with SLE or cutaneous LE the administration of sirukumab led to a dose-independent reduction in the number of leukocytes, neutrophils, and platelets. Treatment was well tolerated; infections and other AEs were only slightly more frequent than in the placebo group [141]. Subsequent phase II proof-of-concept study was conducted in which patients were randomized to receive IV administration of sirukumab (10 mg/kg BW every 4 weeks until week 24) or placebo. The study was carried out on a group of 25 patients with International Society of Nephrology/Renal Pathology Society (ISN/RPS) class III/IV LN and persistent proteinuria despite standard-of-care treatment. Sadly, this trial did not show the expected safety and efficacy of the drug; as many as 48% of the treated patients experienced AEs, mostly infections [142].

## 5. Interleukin-2

IL-2 plays a key role in the activation and stimulation of T cell proliferation [143, 144]. The reduced production of IL-2 in patients with SLE is most likely the underlying cause of pathogenically significant abnormalities, including reduction of counterinflammatory regulatory T cell subset counts and the number of cytotoxic lymphocytes [145]. In a clinical trial performed on a small group of patients with SLE (n = 38) who received low-dose IL-2, almost 90% SRI-4 response rate was demonstrated. In addition, a decrease in the serum levels biomarkers for SLE (complement components, anti-dsDNA antibodies) and proteinuria was observed [146]. However, this promising result needs to be confirmed in a placebo-controlled, blinded study.

## 6. Complement-Targeted Therapies

Early complement components are an important element of immune complexes and apoptotic cells clearance systems in SLE patients. These proteins play an important role in regulating the function of both T and B cells. Terminal complement components activation is associated with flares of the disease and directly responsible for tissue and organ damage, particularly in the course of LN [147].

### 6.1. Eculizumab

Eculizumab is a humanized monoclonal antibody that binds to the C5 complement protein, which prevents its degradation into the active forms of C5a and C5b and the formation of the C5b-9 complex (the alternative pathway for complement activation). This molecule therefore protects against the consumption of early complement components (C1-C4). Eculizumab is registered for the treatment of paroxysmal nocturnal hemoglobinuria and the atypical form of hemolytic-uremic syndrome [148]. In view of promising observations from experimental studies on mouse lupus models, phase I study with a single dose of the drug was undertaken; eculizumab was safe and well tolerated. Notably, the effectiveness of complement blockade was noted for higher doses alone and only in the first 5-10 days of treatment; no improvement in the final stage of the study was observed [149].

## 7. T Cell Costimulation Blockade

### 7.1. Anti-CD40L Antibodies

#### 7.1.1. Ruplizumab and Toralizumab

Ruplizumab is a humanized monoclonal antibody directed against the CD40 ligand (CD40L, CD154) preventing the activation of T cells and the dependent activation of B cells [150]. A clinical trial was conducted with this molecule in LN, which was prematurely terminated due to thromboembolic complications. This serious AE was attributed to the binding of the drug Fc receptor to the platelet IgG Fc IIA receptor (Fc*γ*RIIA) [151]. Preliminary analysis of the treatment results showed a marked decrease of hematuria and proteinuria, as well as the serum level of complement components and anti-dsDNA antibody titres. Administration of toralizumab, a molecule with a similar mechanism of action, was also associated with thromboembolic complications that prevented its further use [152].

#### 7.1.2. Dapirolizumab

Dapirolizumab (known as anti-CD40L Fab-PEG) is a new anti-CD40L antibody that contains a PEGylated Fab' fragment with no Fc portion. For this reason, it is considered safer than its predecessors described above. Phase I clinical trial, lasting 32 weeks, showed a decrease in disease activity in the treatment group. No significant AEs were observed, including thromboembolic complications [153]. It needs to be pointed out that, due to the small number of recruited patients, further evaluation of this improved biologic is required to properly address its efficacy and safety. Recruitment for the phase II trial is in progress [154].

### 7.2. Inhibition of the CD28:CD80/CD86 T Cell Costimulatory Pathway

#### 7.2.1. Abatacept

Abatacept (CTLA4Ig) is a recombinant soluble fusion protein composed of the extracellular domain of the human cytotoxic T-lymphocyte-associated antigen 4 (CTLA-4) and a modified Fc fragment of human Ig 1 (IgG1). Abatacept selectively modulates a key costimulatory signal that is necessary for the full activation of CD28+ T cells. Abatacept has the ability to bind and block CD80 (B7-1) or CD86 (B7-2) molecules on the surface of antigen presenting cells with greater affinity than CD28, inhibiting this key costimulatory signaling pathway for T cell activation. Abatacept inhibits mainly naïve T cell activation and has a much less pronounced effect on inhibiting the activation of memory CD4+ T cells [155]. Studies on mouse models of SLE showed that abatacept delayed the development of autoimmunity and decreased mortality by reducing the number of autoreactive B cells, diminishing Ig class switching and antibody production, as well as reducing the severity of LN [156, 157]. Abatacept and CY combination had a greater effect on disease severity reduction and mortality than any of these drugs used alone in NZB/W mice with LN [158].

In a randomized, double-blind, placebo-controlled phase IIb trial, patients with non-life-threatening SLE whose primary manifestations were active polyarthritis, discoid lupus erythematosus skin lesions, and serositis (pleuritis and/or pericarditis) were receiving abatacept in combination with 30 mg/day prednisone with subsequent tapering 1 month into the therapy. After 12 months of treatment, there were no significant differences in the percentage of patients achieving a primary and secondary end point between the abatacept and placebo groups. However, it was observed that patients with polyarthritis as the main manifestation of the disease achieved the greatest benefits from the therapy. Serious AEs were more frequent in the actively treated group [159].

Another multicentre, randomized, double-blind phase II/III clinical trial that aimed to evaluate the efficacy of the drug was conducted in patients with active LN (ISN/RPS class III/IV). Abatacept was administered in two dosing regimens in combination with prednisone and MMF. At week 52, the composite primary end point, i.e., the time required to achieve complete renal response (urine protein/creatinine ratio <0.26, inactive urinary sediment, and glomerular filtration rate decline not greater than 10% compared to baseline), did not differ significantly between the treatment groups and the placebo group [160].

The previously described efficacy of combined administration of abatacept and CY was the driving force behind the randomized, double-blind, placebo-controlled phase II add-on clinical trial (ACCESS), which enrolled 134 patients with LN. Patients were treated with either abatacept or placebo in conjunction with the Euro-Lupus regimen of low-dose CY followed by azathioprine [161]. At week 24, no statistically significant difference was found in the complete renal response between the abatacept and placebo group (33% vs. 31%). Significant benefits in patients receiving abatacept have also not been observed with respect to partial renal responses or other secondary end points, i.e., reduction of anti-dsDNA antibody levels, normalization of serum concentration of C3 and C4 complement components, shortening of the time needed to achieve complete/partial renal response, improvement of QoL, and frequency of SLE flares. About a half of abatacept-treated patients who achieved complete renal response and, according to the study protocol, discontinued the additional administration of immunosuppressive drugs at week 24 maintained this response by week 52. Treatment was well tolerated [162].

A Phase III, randomized, double-blind, placebo-controlled clinical trial is underway to assess the safety and efficacy of abatacept in patients with active ISN/RPS class III/IV LN treated with MMF and prednisone simultaneously [163].

## 8. Summary

The results of the clinical trials conducted so far with biological drugs in SLE are not encouraging. The only molecule whose use in extrarenal disease is fully justified is belimumab with its modest effect on lupus activity. Despite the promising results of SLE therapy using RTX in cohort observations and numerous reports on the advantages of including this monoclonal antibody in standard therapy, the results of two major clinical trials (EXPLORER and LUNAR) were contrasting with the perceived “real-life” benefits. To the best of the current knowledge, the remaining biotherapies can at most be used in narrow, currently not yet fully identified subsets of patients in terms of clinical and immunological features.

SLE is a disease with a serious prognosis that runs an unpredictable, often life-threatening, course. With these features of lupus in mind, the recruitment of patients with a high severity of disease symptoms due to risk of significant disease progression in the control group or in case of inefficacy of the studied molecule in the treated group is difficult to estimate. Usually, recruitment of patients with fairly low disease activity and/or a less aggressive course is preferred, with the exception of those clinical trials focusing on LN.

A very high clinical heterogeneity of SLE poses major difficulty at the level of design and implementation of clinical trials. The complex nature of SLE makes it thus almost unrealistic to recruit a homogeneous subpopulation of patients. A potentially meaningful solution for overcoming this tendency that could be employed is designing more clinical trials or subtrials with an organ‐specific approach instead of disease activity-specific strategy of recruitment. It would allow obtaining more homogenous population for a trial; however, enrollment of adequate number of patients would be more challenging.

The lack of consistency between the various tools utilized to assess disease activity and different composite indices employed as the primary end points generate discrepancies in the evaluation of patients, hindering the final analysis and interpretation of the clinical trial results. For instance, numerous examples have been provided that the treatment efficacy of patients with LN, a seemingly homogeneous subpopulation, evaluated using different tools led to markedly divergent results [164]. Comprehensive evaluation of the patient, forced by the complex nature of SLE, is not always possible to perform accurately in the clinical trial setting.

In addition to constant attempts to develop new molecules, works on identifying new targets for SLE therapy are equally intense [165]. Despite the above described failures, there has been a significant increase in the number of clinical trials conducted using innovative methods of treatment in patients with SLE in recent years. It remains to be hoped that the extremely dynamic development of basic research and the breakthrough in biological therapy of other autoimmune diseases will finally allow an effective and safe long-term control of SLE in the near future.

## Figures and Tables

**Table 1:** Biologics in systemic lupus erythematosus.

Agent	Mechanism of action	Molecular target	Clinical phase of completed clinical trials	Clinical phase of ongoing clinical trials	Major outcomes/Safety profile	Future prospects
Belimumab	BLyS inhibition	BLyS (soluble)	III [19, 20, 27]	III, IV	Significantly higher SRI-4 response at week 52 in three major phase III clinical trials than placebo. Positive impact on immunological parameters.	++ (approved in EU and US as for the use in non-renal SLE as add-on therapy)

Tabalumab	BLyS/APRIL inhibition	BLyS (soluble and membrane-bound)	III [38, 39]	-	No significant benefits over placebo (SRI-5). Slightly higher proportion of responders compared to placebo in *post hoc* analysis using SRI-4.	+/- (studies were discontinued)

Blisibimod	BLyS/APRIL inhibition	BLyS (soluble and membrane-bound)	III [43]	-	No significant clinical benefits. Beneficial biological effects.	+/-

Atacicept	BLyS/APRIL inhibition	BLyS, APRIL	IIb [49]	-	Slightly better SRI-4 response in patients with low-to-moderate disease activity compared to placebo. High risk of infective complications.	+/-

Abetimus sodium	B cell tolerance induction	Anti-dsDNA antibody-producing B cells/circulating anti-dsDNA antibodies	II/II, III [53, 54]	-	Promising results in phase II/III clinical trials. No efficacy in phase III clinical trials.	+/-

Rituximab	B cell depletion	CD20	III [62, 63]	II, III (including LN)	Primary end points in clinical trials not reached. Large number of reports and recommendations confirming its efficacy in certain subsets of patients.	+

Ocrelizumab	B cell depletion	CD20	III [85]	-	Clinical trials prematurely terminated due to increased risk of adverse events.	-

Ofatumumab	B cell depletion	CD20	Case series [87, 88]	-	Reduction of disease activity and anti-dsDNA antibody titres. Normalization of C3 complement component.	+

Obinutuzumab	B cell depletion	CD20	Preclinical studies [93]	II (LN)	B cell depletion at least 2-fold more efficient than rituximab.	+

Epratuzumab	B cell signaling modulation	CD22	III [102]	-	Lack of clinical efficacy. Favorable safety profile.	-

Rontalizumab	Type I interferon inhibition	IFN*α*	II [119]	-	No general superiority over placebo. Significant benefit in low IFN signature group.	+/-

Sifalimumab	Type I interferon inhibition	IFN*α*	IIb [122]	-	Better SRI-4 response in high IFN signature group.	+ (studies were discontinued)

Anifrolumab	Type I interferon inhibition	IFNAR1	IIb [126]	III (several trials including LN)	Better SRI-4 response in high IFN signature group.	+

Tocilizumab	Cytokine inhibition	IL-6 receptor	I [140]	-	Improvement in clinical parameters. Reduction of anti-dsDNA antibody titers.	+

Sirukumab	Cytokine inhibition	IL-6	II (proof-of-concept) [142]	-	Lack of clinical efficacy. Frequent serious adverse events.	-

Eculizumab	Complement blockade	Monoclonal antibody against complement component C5	I [149]	-	Short-lasting biological efficacy was noted only for higher doses.	-

Ruplizumab/ Toralizumab	T cell costimulation blockade	CD40L	Open label/ I [151, 152]	-	Clinical trials were terminated due to thromboembolic complications.	-

Dapirolizumab	T cell costimulation blockade	CD40-CD40L	Ib [153]	III	Disease activity reduction (SRI-4, BICLA).	+

Abatacept	T cell costimulation blockade	CD28/CTLA4-CD80/CD86	IIb, II/III [159, 160]	III (including LN)	No significant clinical benefits. Most beneficial in patients who had polyarthritis as the primary manifestation. Safe and well tolerated.	+

APRIL: a proliferation-inducing ligand; BICLA: BILAG-based Combined Lupus Assessment; BLyS: B lymphocyte stimulator; CD40L: CD40 ligand; CTLA4: cytotoxic T lymphocyte associated protein 4; IFN*α*: interferon alpha; IFNAR1: interferon alpha receptor 1; IL-2: interleukin 2; IL-6: interleukin 6; LN: lupus nephritis; SLE: systemic lupus erythematosus; SRI-4: Systemic Lupus Erythematosus Responder Index 4.
